# Results of consecutive training procedures in pediatric cardiac surgery

**DOI:** 10.1186/1749-8090-5-105

**Published:** 2010-11-08

**Authors:** Serban C Stoica, David N Campbell

**Affiliations:** 1 Dept. of Pediatric Cardiac Surgery, Bristol Heart Institute and Children's Hospital, Bristol, UK; 2 Dept. of Pediatric Cardiac Surgery, Children's Hospital, Denver, Colorado, USA

## Abstract

This report from a single institution describes the results of consecutive pediatric heart operations done by trainees under the supervision of a senior surgeon. The 3.1% mortality seen in 1067 index operations is comparable across procedures and risk bands to risk-stratified results reported by the Society of Thoracic Surgeons. With appropriate mentorship, surgeons-in-training are able to achieve good results as first operators.

## Background

Congenital heart surgery evolved from experimental life-saving operations to treatment algorithms, risk stratification and quality control. This environment challenges the transfer of skills to new recruits. A variety of perceptions may hamper training: time or team constraints, procedure complexity, trainee's ability, trainer's commitment, lack of 'chemistry' between mentor and apprentice, patient's family demands or a combination of these. Many talented surgeons have learned 'by osmosis', through closely assisting an expert. If one gets better by performing rather than seeing a task, then regardless of aptitude it is preferable to progress from assistant to operator while still a trainee. To reduce the variability in exposure the newly developed certificate of congenital training in the US has strict requirements for the number and types of primary surgeon cases [1]. We report in this context the results of a pediatric attending (DNC) with special interest in training.

## Patients and Methods

Whenever a trainee is available it has been the senior author's policy that he/she is the primary surgeon, remaining on the operator's side throughout the case. We do not have surgical practitioners. (Procedures done at a non-academic institution as well as congenital cases done at the adult university hospital are not reported here because of lacking risk stratification in these databases. Training however was the same. At the adult university hospital the practice consists of the full range of adult congenital disease and ductal ligations in the maternity, all of which became training cases for residents on service.) The current report therefore includes 1443 consecutive operations done under supervision by 7 fellows at Denver Children's Hospital between January 2003, when the Aristotle Basic Complexity score (ABCS) was introduced, and May 2009. In 33 cases where a trainee was not available another attending operated with the senior author assisting. Recently there was a change in referral patterns, the senior author taking responsibility for the Norwood program, and 6 stage I operations became 2-attending procedures. These are the only non-training cases in the series, leaving 1404 operations for analysis. To concentrate further on main procedures, after exclusion of chest reopening, delayed closure, pacemaker and patent ductus operations, wound and drainage procedures, but including chylothorax operations, 1067 index training cases were retained (Table 1). A comparison of their risk profile with that of the 33 non-Norwood 2-attending cases suggested no selection bias (ABCS, 7.1 ± 2.0 vs. 7.3 ± 2.2, p = 0.60, t test). 435 procedures (40.7%) were in the levels 3 and 4 of complexity (ABCS ≥8.0). The operative mortality for the 1067 index cases, defined by registry criteria [2], was 33 (3.1%).

**Table 1:** Patient details for 1067 index training cases

**Age **(years), median (interquartile range) (range)		0.7 (0.2, 7.1) (0.0, 44.1)		
**Weight **(kg), median (interquartile range) (range)		6.9 (3.9, 20.6) (0.9, 178.2)		

**Basic Aristotle Score**, mean (standard deviation) (range)		7.1 (2.0) (1.5, 14.5)		

**Procedure**	**N**	**Hospital mortality (%)**	**Discharge % mortality STS database **[3]	**Late mortality (%)**^**a**^

Coarctation of the aorta, arch surgery, aortic aneurysm	148	5 (3.4)	N/a	0

Ventricular septal defect (incl. 1 hybrid perventricular)	133	0	0-1.1	0

Heart transplantation	81	5 (6.2)	6.0	2 (2.5)

ECMO cannulation/decannulation	72	5 (6.9)	N/a	4 (5.5)

Right ventricular outflow procedure	69	0	4-5.8	0

Atrio-ventricular canal	57	0	1.3, 4.5^b^	0

Atrial septal defect	39	0	1.4	0

Tetralogy of Fallot repair	39	1 (2.5)	0.4-2.7	0

Systemic to pulmonary shunt	35	4 (11.4)	7.6	1 (2.8)

Glenn	35	0	2	0

Vascular ring/sling	29	1 (3.4)	N/a	0

Fontan (incl. 2 conversions)	27	1 (3.7)	3.9	0

Pericardial procedure	27	0	N/a	0

Ross, Konno, Ross-Konno	24	2 (8.3)	2.3^c^	0

Mitral valve replacement	20	2 (10)	N/a	0

Pulmonary artery banding debanding	17	0	N/a	0

Aortic stenosis sub-/supravalvar	17	0	0^d^	0

Partial anomalous pulmonary venous drainage	15	0	N/a	0

Pleural drainage/decortication	14	0	N/a	0

Pectus procedure	13	0	N/a	0

Total anomalous pulmonary venous drainage	12	1 (8.3)	9.0	0

Diaphragm plication	11	0	N/a	0

Aortic root replacement (incl. 5 valve-sparing)	11	0	N/a	0

Aortic valve replacement	10	0	N/a	0

Truncus arteriosus	8	2 (25)	N/a	0

Tricuspid valve procedure	7	0	N/a	0

Pulmonary artery reconstruction	7	1 (14.3)	N/a	0

Coronary procedures	7	0	N/a	0

PA-VSD procedure	6	0	N/a	0

Mitral valve repair	6	1 (16.6)	1.4	0

Norwood stage I	6	0	31.4	1 (16.6)

Pulmonary valve/Right ventricular outflow tract enlargement	5	0	N/a	0

Cor triatriatum, supravalvar mitral ring	4	0	N/a	0

Double chambered right ventricle	4	0	N/a	0

Ventricular assist device (excl. transplantation)	3	1 (33.3)	N/a	0

Atrial septal defect creation/enlargement	3	0	N/a	0

Aortic valve repair	3	0	N/a	0

Arterial switch	2	0	2.0	0

Rastelli	2	0	N/a	0

Double outlet right ventricle, intraventricular tunnel	2	0	N/a	0

Aorto-pulmonary window	1	0	N/a	0

Pulmonary vein stenosis	1	0	N/a	0

One-and-a-half ventricle repair	1	0	N/a	0

Mustard	1	0	N/a	0

Other	33	0		0

Total	1067	33 (3.1)		7 (0.6)

N/a, not available; a - in addition to early mortality; b - for partial and complete AV canal respectively; c - for Ross operation; d - for subvalvar aortic stenosis

## Discussion

Congenital training arrangements are summarized by Kogon's recent survey of 11 large programs, with 28 of 42 trainees responding (67%) [1]. Encouragingly, the vast majority were satisfied with training overall however only 10 were satisfied with the operative experience. Each fellow performed a mean of 75 (± 53) operations and 51 (± 42) open cases - note the variability. The majority did not perform any operations in the higher complexity range, as defined by a Risk Adjusted Congenital Heart Surgery Score of 4-6. The perception remains that apprenticeship, particularly for complex cases, continues even after training is over. We agree this is a reasonable expectation.

This report shows that the congenital operative experience can be maximized. All training deterrents enumerated in the introduction were consistently neutralized. By including consecutive patients and trainees selection bias is eliminated. Despite a significant number of complex cases the early outcomes were good, comparable with reports from the Society of Thoracic Surgeons [3] (Table 1). Our conclusion is limited by the absence of prospectively collected data to demonstrate that morbidity, but also cost and long-term results are not affected. However, another study in adults showed that training and non-training cardiac cases have similar long-term outcomes [4]. In summary, operative training is possible in consecutive congenital cases without increased risk to patients. We do not advocate a blanket adoption of this by other teams. It should be attempted only when everybody is comfortable and, above all, never at the patients' expense.

## Competing interests

The authors declare that they have no competing interests.

## Authors' contributions

SCS and DNC wrote the paper, DNC is the program director and supervised the training of residents as described. Both authors read and approved the final manuscript.
